# Prognostic implications of invasive hemodynamics during cardiac resynchronization therapy: Stroke work outperforms dP/dt_max_

**DOI:** 10.1016/j.hroo.2023.11.003

**Published:** 2023-11-10

**Authors:** Philippe C. Wouters, Alwin Zweerink, Wouter M. van Everdingen, Mohammed A. Ghossein, Gerben J. de Roest, Maarten J. Cramer, Pieter A.F.M. Doevendans, Kevin Vernooy, Frits W. Prinzen, Cornelis P. Allaart, Mathias Meine

**Affiliations:** ∗ Department of Cardiology, UMC Utrecht, Utrecht, the Netherlands; † Department of Cardiology, Amsterdam UMC, Amsterdam, the Netherlands; ‡ Department of Radiology and Nuclear Medicine, Rijnstate Hospital, Arnhem, the Netherlands; § Department of Cardiology, Cardiovascular Research Institute Maastricht, Maastricht University Medical Center+, Maastricht, the Netherlands; || Department of Cardiology, St. Antonius, Nieuwegein, the Netherlands; ¶ Netherlands Heart Institute, Utrecht, The Netherlands; ∗∗ Department of Physiology, Cardiovascular Research Institute Maastricht, Maastricht University, Maastricht, the Netherlands

**Keywords:** Cardiac resynchronization therapy, CRT, Stroke work, Prognosis, dPdt_max_

## Abstract

**Background:**

Invasive measurements of left ventricular (LV) hemodynamic performance can evaluate acute response to cardiac resynchronization therapy (CRT).

**Objective:**

The study sought to determine which metric, maximum rate of LV pressure rise (LV dP/dt_max_) or LV stroke work (LVSW), is more strongly associated with long-term prognosis.

**Methods:**

CRT patients were prospectively included from 3 academic centers. Invasive pressure-volume loop measurements during implantation were performed, and LV dP/dt_max_ and LVSW were determined at baseline and during biventricular pacing (BVP) as well as their relative increase (%Δ). Hazard ratios (HRs) for the primary outcome of 8-year all-cause mortality were derived using Cox proportional hazards. The secondary endpoint was echocardiographic response, defined as 6-month LV end-systolic volume reduction ≥15%.

**Results:**

Paired data from 82 patients were analyzed (67% male; age 66 ± 9 years; QRS duration 158 ± 22 ms, median survival time 72 months). Survival was better when LVSW during BVP was ≥4400 mL∙mm Hg (HR 0.21, 95% CI 0.08–0.58, *P* < .003) or when ΔLVSW% was ≥10% (HR 0.22, 95% CI 0.08–0.65, *P* = .006). In multivariate analysis, following direct comparison of continuous measures of acute ΔLV dP/dt_max_% and ΔLVSW%, only ΔLVSW% remained associated with the primary endpoint (HR 0.982 per percentage point, *P* = .028). In contrast to LV dP/dt_max_ (all *P* > .05), significant associations with echocardiographic response were found for stroke work during BVP (area under the receiver-operating characteristic curve 0.745, *P* = .001) and ΔLVSW% (area under the receiver-operating characteristic curve 0.803, *P* < .001).

**Conclusion:**

Stroke work, but not LV dP/dt_max,_ is consistently associated with long-term prognosis and response after CRT. Our results therefore favor the use of stroke work as the hemodynamic parameter to predict long-term outcome after CRT.

## Graphical abstract

**Figure:**
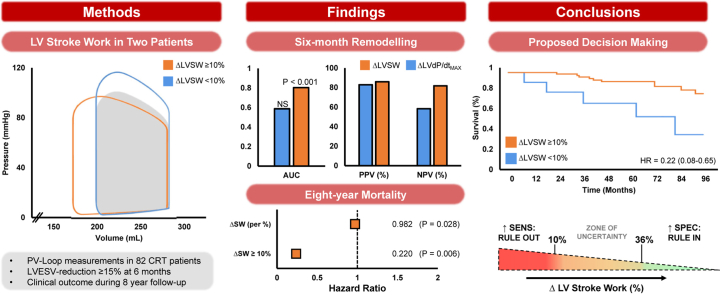

**Key Findings**

▪ Change in maximum rate of left ventricular (LV) pressure rise and change in LV stroke work (ΔLVSW%) cannot be used interchangeably.
▪ A ≥10% acute increase in LVSW after cardiac resynchronization therapy was associated with a 4-fold survival benefit during 8-year follow-up, independent of baseline LVSW.
▪ Following direct comparison of change in maximum rate of LV pressure rise and ΔLVSW%, only the latter remained significantly associated with clinical outcome.
▪ Our results favor the use of LVSW as best hemodynamic parameter, for example to evaluate the effect of LV lead position and pacing delays during cardiac resynchronization therapy optimization.

## Introduction

Invasive measurements of left ventricular (LV) hemodynamic performance can be used to optimize cardiac resynchronization therapy (CRT). For instance, acute change in maximum rate of LV pressure rise (LV dP/dt_max_) can be used to optimize device settings^1^, ^2^, ^3^ or help navigate the LV lead to hemodynamically optimal positions during implantation.^4^, ^5^, ^6^ However, acute changes in LV dP/dt_max_ do not necessarily predict clinical outcome.^7^ Alternatively, pressure-volume loop measurements using a conductance catheter can be performed to derive LV stroke work (LVSW). LVSW favors LV–arterial coupling and mechanical efficiency, rather than LV isovolumetric contractility, and is more strongly associated with LV reverse remodeling and LV ejection fraction improvement than LV dP/dt_max_.^8^^,^^9^ Whether LVSW is also associated with long-term outcomes remains to be investigated. We sought to directly compare the acute improvement in LV dP/dt_max_ and LVSW in heart failure patients with a de novo CRT implantation for their association with all-cause mortality and echocardiographic response.

## Methods

As part of 2 previously conducted studies,^8^^,^^10^ a total of 92 de novo CRT implants in patients with symptomatic heart failure and a LV ejection fraction ≤35% were prospectively included from 3 academic centers in the Netherlands, according to the CRT guideline indication at the time.^11^ Left bundle branch block (LBBB) morphology was scored according to the Strauss criteria. The definite LV lead position was determined based on the absence of phrenic nerve stimulation and presence of acceptable pacing thresholds. An anterior or posterior LV lead position was avoided when possible, but precise lead positions were not systematically assessed. Ultimately, the location that resulted in either the best acute hemodynamic benefit or intrinsic electrical delay, as measured through Q-LV sense, was chosen.^8^^,^^10^ All patients were in sinus rhythm, and the pacing rate was kept constant at 5 to 10 beats/min above intrinsic rhythm. Although no uniform pacing delays were mandated across both studies, atrioventricular delays were set at 100 ms sensed or 120 ms paced, and interventricular delays at 0 or 40 ms LV preactivation.^8^^,^^10^ During implantation, invasive pressure-volume loop measurements were performed. Echocardiography was performed at baseline and at 6-month follow-up. The primary outcome was 8-year all-cause mortality. The secondary endpoint was echocardiographic response, defined as 6-month LV end-systolic volume reduction ≥15%. The study was performed according to the Declaration of Helsinki and received approval of the local medical ethics committees. All subjects gave written informed consent.

### Hemodynamic measurements

Invasive pressure-volume loop measurements were performed, and LV dP/dt_max_ and LVSW as distinct measures of hemodynamic performance were calculated. LVSW was calculated as the area of the pressure-volume loop, thereby estimating the product of stroke volume and mean arterial pressure during LV ejection. To this end, a 7-F conductance catheter (CD Leycom, Zoetermeer, the Netherlands) was inserted via the femoral artery and the tip of the catheter was placed in a stable position in the LV apex. Baseline measurements (ie without pacing) were taken before and after biventricular pacing (BVP). Data were acquired during baseline conditions and 30 seconds after initiating pacing. Approximately 60 representative cardiac cycles were averaged, excluding all inappropriate beats (ie extrasystoles). For this study, measurements at the electrode selected for chronic pacing were performed at baseline and during BVP. In addition, acute relative changes in either LV dP/dt_max_ and LVSW were calculated (Δ%). Patients were excluded in case of nonanalyzable baseline loops (ie nonphysiologic volume changes during the isovolumic phase), as characterized by hourglass-shaped loops, following independent judgement by 2 experts (P.C.W. and A.Z.).

### Statistics

Hazard ratios (HRs) for the primary outcome of 8-year all-cause mortality were derived using Cox proportional hazards. Separate models were created, for either LV dP/dt_max_ or LVSW as a continuous variable in incremental steps of 100 mm Hg/s or 100 mL·mm Hg, respectively. Optimal time-dependent dichotomized values were used to derive survival curves using the Kaplan-Meier method for each hemodynamic parameter. The secondary endpoint was volumetric response, defined as a 6-month LV end-systolic volume reduction of ≥15%. This endpoint allows for intrapatient comparison and is commonly used as a surrogate marker of clinical outcome.^12^ The area under the receiver-operating characteristic curve (AUROC) was used to determine the association of hemodynamic performance with echocardiographic response. A *P* value of <.05 was considered significant. Data analysis was performed with SPSS 25.0 (IBM Corporation, Armonk, NY).

## Results

### Study population

Ten patients demonstrated hourglass shaped pressure-volume loops and were excluded from further analysis. These loops typically occurred in patients with significantly longer QRS duration, larger end-diastolic volume, larger end-systolic volume , and lower LV ejection fraction (*P* < .01 for all). Of note, by itself, hourglass-shaped measurements were significantly associated with all-cause mortality (*P* = .025). In the remaining 82 patients (67% male; age 66 ± 9 years; 42% New York Heart Association functional class II; QRS duration 158 ± 22 ms), suitable acute invasive measurements of LV dP/dt_max_ and LVSW with long-term follow-up and paired echocardiographic examinations were available (Table 1). When stratified according to echocardiographic response, QRS duration and LBBB morphology were significantly different (Table 1). QRS duration correlated weakly with ΔLVSW% (R = 0.321, *P* = .003) and moderately with ΔLV dP/dt_max_% (R = 0.440, *P* < .001). During 8-year follow-up, 15 (20%) deaths occurred. Median survival time was 72 months (interquartile range 59-96 months).

**Table 1:** Patient characteristics at baseline, stratified to echocardiographic response

Parameter	Total (n = 82)	Responders (n = 63)	Nonresponders (n = 19)	*P* value
Male	55 (67)	42 (67)	13 (68)	1.000
Age, y	66 ± 9	66 ± 10	67 ± 6	.560
Non-ICM	52 (62)	40 (64)	11 (58)	.788
NYHA functional class II	34 (42)	28 (44)	6 (32)	.132
QRS duration, ms	158 ± 22	163 ± 19	143 ± 24	<.001
LBBB	60 (79)	51 (86)	9 (53)	.006
β-blocker	61 (74)	48 (76)	13 (68)	.553
ACE inhibitor/ARB	74 (90)	56 (89)	18 (95)	.674
Spironolactone	42 (51)	33 (56)	9 (47)	.601
LVEDV, mL	207 ± 55	210 ± 55	195 ± 55	.266
LVESV, mL	152 ± 51	155 ± 50	142 ± 52	.321
LVEF, %	28 ± 7	28 ± 7	28 ± 7	.953

ACE = angiotensin-converting enzyme; ARB = angiotensin II receptor blocker; EDV = end-diastolic volume; EF = ejection fraction; ESV = end-systolic volume; ICM = ischemic cardiomyopathy; LBBB = left bundle branch block; LV = left ventricular; LVEDV = left ventricular end-diastolic volume; LVESV = left ventricular end-systolic volume; NYHA = New York Heart Association.

### Disagreement in acute hemodynamic parameters

There was no correlation between continuous measures of ΔLVSW% and ΔLV dP/dt_max_% (R = 0.178, *P* = .110). When dichotomized, disagreement in hemodynamic response classification according to ΔLVSW ≥10% and ΔLV dP/dt_max_ ≥0% occurred in 18% of all patients (Cohen's κ = 0.242). Isolated LVSW response was observed in 8 patients (ΔLVSW = 31%; ΔLV dP/dt_max_ = –5%), whereas isolated LV dP/dt_max_ response occurred in 7 patients (ΔLV dP/dt_max_ = 17%; ΔLVSW = –4%).

### Clinical outcome

**Figure 1:**
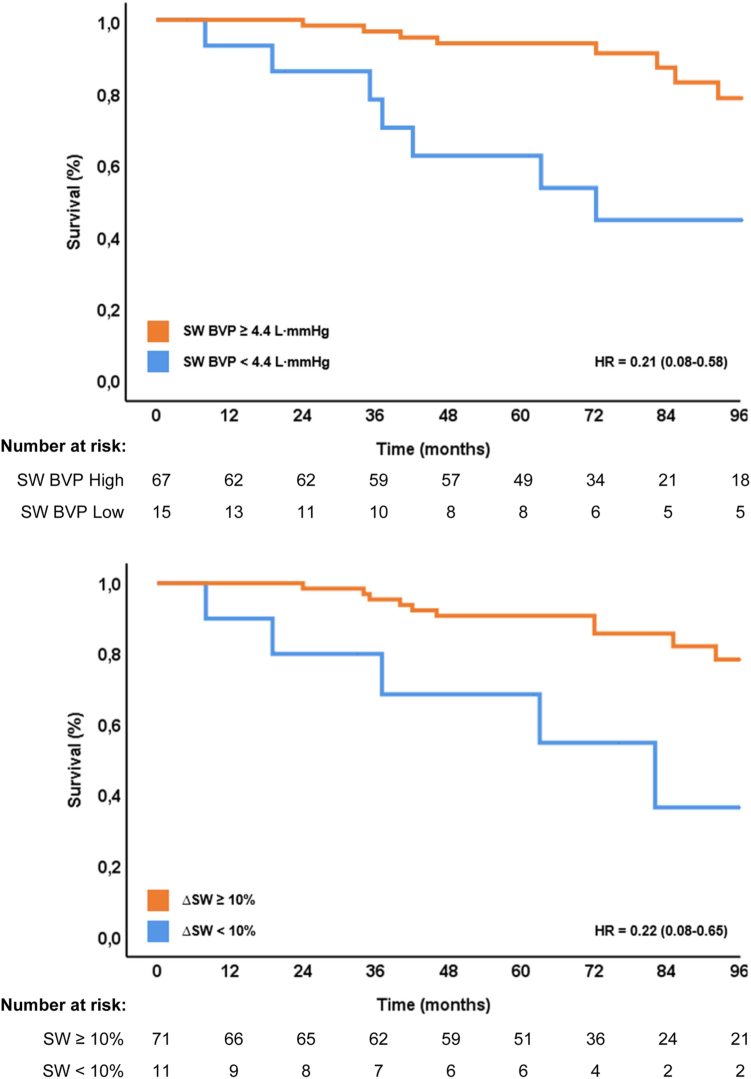
Kaplan-Meier curves for all-cause mortality, stratified according to left ventricular stroke work (SW) during biventricular pacing (BVP) (top), and acute change in left ventricular SW (bottom). HR = hazard ratio.

Following univariate analysis, LV dP/dt_max_ as a continuous variable was never associated with all-cause mortality during 8-year follow-up (Supplemental Table 1). However, only when dichotomized using the optimal cutoff value for clinical outcome and response (Supplemental Table 2, Supplemental Figure 1) was ΔLV dP/dt_max_% ≥0% associated with better 8-year clinical outcome (HR 0.282, 95% CI 0.10–0.80, *P* = .017). By contrast, LVSW during BVP and ΔLVSW% were both identified as significantly associated with all-cause mortality when used as continuous variables (Table 2). In multivariate analysis, directly comparing ΔLV dP/dt_max_% and ΔLVSW%, only the latter remained significantly associated with the primary endpoint, irrespective of baseline values of LVSW or QRS duration (HR 0.98 per percentage point, 95% CI 0.97–0.99, *P* = .028). Kaplan-Meier analysis revealed significant better freedom from all-cause mortality when LVSW during BVP was ≥4400 mL·mm Hg (HR 0.21, 95% CI 0.08–0.58, *P* < .003). The same association was seen for ΔLVSW% ≥10%, also when corrected for baseline LVSW or QRS duration (HR 0.22, 95% CI 0.08–0.65, *P* = .006) (Figure 1). Stroke work responders had significantly longer baseline QRS duration (160 ± 21 ms vs 146 ± 27 ms, *P* = .049) but were proportional similar concerning LBBB morphology (Supplemental Table 3). HRs for LVSW and outcome were unaffected after correction for QRS duration (Table 2). Upon BVP, LV dP/dt_max_ decreased in 12 (15%) patients and LVSW decreased in 6 (7%) patients.

**Table 2:** Cox proportional hazards ratios for stroke work and all-cause mortality within 8 years after cardiac resynchronization therapy

	Left ventricular stroke work	Univariate HR	*P* value
Baseline	Continuous (100 mL·mm Hg)	0.985	.212
	Dichotomous ≥2800 mL·mm Hg	0.613	.374
BVP	Continuous (100 mL·mm Hg)	0.984	**.049**
	Dichotomous ≥4400 mL·mm Hg	0.212	**.003**
Acute Δ	Continuous	0.982	**.028**
	Dichotomous ≥10%	0.220	**.006**

Results and significance were identical when corrected for QRS duration. Values for maximum rate of left ventricular pressure rise can be found in Supplemental Table 1.

BVP = biventricular pacing; HR = hazard ratio.

Bold numbers are statistically significant values.

### Echocardiographic response

**Figure 2:**
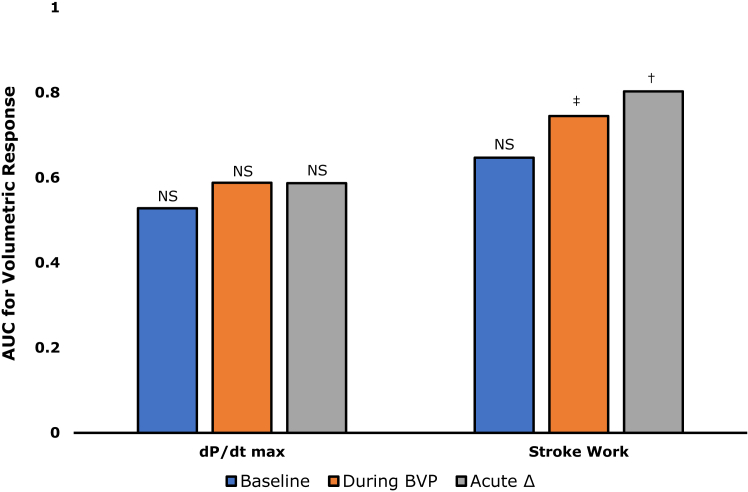
Association of left ventricular (LV) hemodynamic performance, as measured using maximum rate of LV pressure rise (dP/dt_max_) or LV stroke work, and 6-month reverse remodeling. †*P* < .001; ‡*P* = .002. AUC = area under the receiver-operating characteristic curve; BVP = biventricular pacing; NS = nonsignificant.

There were 19 (23%) volumetric nonresponders. No significant association between LV dP/dt_max_ and echocardiographic response was found, independent of whether values at absolute baseline, during BVP, or acute relative changes upon BVP were used (Figure 2). By contrast, LVSW during BVP (AUROC 0.745, 95% CI 0.61–0.88, *P* = .001) and ΔLVSW% (AUROC 0.803, 95% CI 0.68–0.92, *P* < .001) were significantly associated with echocardiographic response (Supplemental Table 2). The association of ΔLVSW% with echocardiographic response was significantly stronger when compared with ΔLV dP/dt_max_% (ΔAUROC 0.216, *P <* .05). A cutoff value of ≥10% for ΔLVSW% was optimal and yielded high sensitivity (Supplemental Table 2). Using this cutoff value, concordance between acute hemodynamic response and 6-month echocardiographic response was achieved in 70 (85%) of 82 patients. Reduction in LV end-systolic volume was significantly associated with 8-year all-cause mortality (HR 0.975 per 1% reduction, *P* = .012). As a result, better 8-year survival (HR 0.35, *P* = .044) was seen in echocardiographic responders.

## Discussion

For the first time, the association between LVSW and long-term clinical outcome is demonstrated. A ≥10% acute increase in LVSW after CRT was associated with a 4-fold survival benefit during 8-year follow-up after CRT, independent of baseline LVSW or QRS duration. In contrast to LV dP/dt_max_, these findings were found consistently, both during BVP and ΔLVSW%, also when expressed as a continuous variable. The association of LVSW and intrapatient reverse remodeling, predictive of good clinical outcome,^12^ provides further support of the mechanistic implications of hemodynamic optimization.

### Evaluating acute hemodynamic performance

Our findings extend the work of Bogaard and colleagues,^7^ who found that baseline and BVP LV dP/dt_max_, but not acute changes, were associated with clinical outcome. However, their study had a short follow-up of only 1 year after CRT and did not evaluate LVSW. Because we investigated up to 8 years of follow-up, our results better reflect true long-term outcome. Our findings suggest that each increase in acute ΔLVSW% is important, but that this may not per se result in a clinically meaningful benefit when LV hemodynamic performance remains poor during BVP (ie below 4400 mL·mm Hg). A cutoff <10% for acute ΔLVSW% had high positive predictive value and negative predictive value and may be suitable to reliably exclude volumetric response and good 8-year clinical outcome. Moreover, a minority of patients experienced decreased hemodynamic performance during CRT, which underscores that CRT can indeed be detrimental in selected cases.^13^ Future studies may investigate whether refraining from BVP in these selected cases is beneficial.

One explanation for the stronger association with outcome when comparing LVSW with LV dP/dt_max_ lies in its physiological background. LVSW reflects the amount of constructive LV mechanical energy that is used to propel a certain blood volume during LV ejection. LVSW thereby integrates information on preload, afterload, and contractility, and is related to myocardial oxygen consumption.^14^ By comparison, LV dP/dt_max_ simply reflects the isovolumetric contraction phase. The present study conformed the discordance of ΔLVSW% and ΔLV dP/dt_max_%, indicating that both measures cannot be used interchangeably.^15^ As a result, use of LV dP/dt_max_ as an optimization target was shown to inadvertently preclude LVSW increase after CRT.^9^

### Noninvasive estimations of LVSW

Acute ΔLVSW% upon CRT is a direct consequence of mechanical recoordination that follows immediately upon adequate correction of septal-to-lateral electrical activation delay.^16^^,^^17^ As a noninvasive alternative to pressure-volume loop measurements (ie LVSW), pressure strain (ie LV myocardial work) measurements can also be performed in the acute setting and have been shown to be related to a volumetric response as well.^17^ Using this approach, however, assumptions on LV pressure are made using brachial artery cuff pressure, rather than by performing actual real-time measurements of hemodynamic changes before and after enabling CRT. Moreover, LV strain requires high-quality acoustic views that necessitate acquisition in the left lateral supine position. This precludes its use for continuous hemodynamic monitoring of LV systolic function in the operating theater, for example during optimization procedures of CRT.

### Clinical implications

Because the optimal pacing site is highly variable, challenging to predict, and patient-specific, stroke work may be used to help navigate the LV lead to the optimal target that results in better outcome.^4^, ^5^, ^6^ In addition, LVSW can be used to evaluate the hemodynamically optimal atrioventricular and interventricular pacing configuration, which is patient specific as well.^1^, ^2^, ^3^^,^^5^ However, because hemodynamic measurements were not formally compared as optimization strategies, a randomized study is needed to investigate potential superiority of either mechanism. Alternative approaches to determine the optimal LV lead position have been proposed, using either electrical or image-based guidance.^18^^,^^19^ While less invasive, these methods rely on indirect mechanisms to provide feedback, rather than evaluate actual changes in LV performance. Therefore, such methods may be less suitable to evaluate device programming or perioperatively compare the effects of different pacing modalities, as is the case when comparing BVP and conduction system pacing. Although the minimally invasive nature of LVSW may limit its use to a selection of patients, it is conceivable that the utility of LVSW extends to a broad range of clinical scenarios. Measuring LVSW through LV catheterization can be useful to monitor patients that are critically ill, require inotropy or mechanical circulatory support, or undergo major cardiac surgery.^14^

### Limitations

Our results should be interpreted in light of all the limitations inherently associated with a nonrandomized design. Because of limited sample size and low number of events, no extensive multivariate adjustment could be performed. A larger prospective trial should define definitive cutoff values. Regardless, the use of 2 similar cohorts allowed for a relatively large sample size considering the invasive hemodynamic nature of the present study (Supplemental Table 4), and patient characteristics reflected a typical CRT population. Unfortunately, in one cohort, data concerning major adverse events such as heart failure hospitalization were not systematically gathered and could not be retrieved. Although conductance catheter pressure signals are reliable, volume signals are more dependent on careful catheter placement. The optimal cutoff for ΔLVSW% and long-term outcome was relatively low at 10%, and only 13% of patients failed to meet this requirement. However, this value coincides with the relatively low number of volumetric nonresponders (23%) and deaths (20%) observed in our study. In addition, this value was found to be optimal in excluding a meaningful benefit from CRT, which is considered the preferred selection approach in order to justify withholding CRT.^20^ Last, future studies should evaluate whether the present findings can be generalized to conduction system pacing.

## Conclusion

Acute increases in LVSW after CRT were associated with a significant survival benefit during 8-year follow-up after CRT. The present study provides new evidence in support of ΔLVSW% as the hemodynamic parameter that best reflects structural response and clinical outcome in CRT patients.
