# Supplementary material for: Prognostic implications of invasive hemodynamics during cardiac resynchronization therapy: Stroke work outperforms dP/dtmax
Source: Heart Rhythm O2. 2023 Nov 10;4(12):777–83. doi: 10.1016/j.hroo.2023.11.003 (PMC10774665; doi:10.1016/j.hroo.2023.11.003)
Supplement: Supplementary Data [file mmc1.docx]

**Appendices**

***Supplemental Table 1.*** *Cox proportional hazard ratio’s for LVdP/dt_MAX_ and all-cause mortality within 8 years after cardiac resynchronization therapy.*

|  | **LVdP/dt_MAX_** | **Univariate HR** | **P-value** |
| --- | --- | --- | --- |
| **Baseline** | Continuous (100 mmHg/s) | 0.598 | 0.940 |
|  | Dichotomous ≥ 620 mmHg/s | 0.399 | 0.096 |
| **BVP** | Continuous (100 mmHg/s) | 0.827 | 0.156 |
|  | Dichotomous ≥ 720 mmHg/s | 0.427 | 0.121 |
| **Acute ∆** | Continuous (%) | 0.977 | 0.258 |
|  | Dichotomous ≥ 0 % | 0.282 | **0.017** |

*Legend: BVP, biventricular pacing; HR, hazard ratio.*

***Supplemental Table 2.*** *Test characteristics of optimal cut-off values associated with 6-month volumetric response.*

| **Predictor** | **AUC** | **p-value** | **Cut-off value** | **Sens (%)** | **Spec (%)** | **PPV (%)** | **NPV (%)** |
| --- | --- | --- | --- | --- | --- | --- | --- |
| **LVSW** |  |  |  |  |  |  |  |
| Baseline | 0.647 | 0.052 | ≥ 2600 mL∙mmHg | 90.5 | 42.1 | 83.3 | 57.1 |
| BVP | 0.745 | **0.001** | ≥ 5600 mL∙mmHg | 73.0 | 73.7 | 88.5 | 43.3 |
| Acute ∆ | 0.803 | **< 0.001** | ≥ 10% | 95.2 | 52.6 | 85.9 | 81.8 |
| Acute ∆ | 0.803 | **< 0.001** | ≥ 36% | 69.8 | 78.9 | 89.8 | 42.4 |
| **LVdP/dt_MAX_** |  |  |  |  |  |  |  |
| Baseline | 0.528 | 0.709 | ≥ 760 mmHg/s | 65.1 | 52.6 | 80.4 | 29.0 |
| BVP | 0.588 | 0.246 | ≥ 780 mmHg/s | 77.8 | 47.4 | 83.3 | 40.9 |
| Acute ∆ | 0.587 | 0.253 | ≥ 0% | 92.1 | 36.8 | 82.9 | 58.3 |

*Legend: AUC, area under the curve; BVP, biventricular pacing; LVSP, Left ventricular stroke work; NPV, negative predictive value; PPV positive predictive value; Sens, sensitivity; Spec, specificity.*

***Supplemental Table 3.*** *Differences in patient characteristics between Stroke Work response.*

| **Parameter** | **SW <10% (n=11)** | **SW ≥10% (n=71)** | **P-value** |  |
| --- | --- | --- | --- | --- |
| Male sex – n (%) | 11 (100) | 44 (62) | **0.013** |  |
| Age (years) | 67±5 | 66±10 | 0.198 |  |
| Non-ICM – n (%) | 5 (46) | 46 (65) | 0.317 |  |
| NYHA II – n (%) | 5 (46) | 29 (41) | 0.895 |  |
| QRS duration (ms) | 146±27 | 160±21 | **0.049** |  |
| LBBB – n (%) | 6 (60) | 54 (82) | 0.204 |  |
| β-blocker – n (%) | 52 (73) | 9 (82) | 0.720 |  |
| ACEi/ARB – n (%) | 64 (90) | 10 (91) | 1.000 |  |
| Spironolactone – n (%) | 2 (18) | 40 (60) | **0.019** |  |
| LVEDV (ml) | 201±61 | 208±54 | 0.706 |  |
| LVESV (ml) | 145±59 | 153±50 | 0.621 |  |
| LVEF (%) | 29±8 | 28±7 | 0.688 |  |

*Legend: ACEi, angiotensin-converting-enzyme inhibitor; ARB, angiotensin-II receptor blocker; EDV, end-diastolic volume; EF, ejection fraction; ESV, end-systolic volume; ICM, ischemic cardiomyopathy; LBBB, left bundle branch block; LV, left ventricular; NYHA, New York Heart Association.*

***Supplemental Table 4.*** *Differences in patient characteristics between study groups.*

| **Parameter** | **OPTICARE (n=41)** | **TBS (n=41)** | **P-value** |  |
| --- | --- | --- | --- | --- |
| Male sex – n (%) | 25 (61) | 30 (73) | 0.372 |  |
| Age (years) | 67±9 | 65±10 | 0.521 |  |
| Non-ICM – n (%) | 33 (81) | 18 (44) | **0.001** |  |
| NYHA II – n (%) | 29 (71) | 5 (12) | **<0.001** |  |
| QRS duration (ms) | 167±14 | 150±25 | **<0.001** |  |
| LBBB – n (%) | 41 (100) | 19 (54) | **<0.001** |  |
| β-blocker – n (%) | 31 (76) | 30 (73) | 1.000 |  |
| ACEi/ARB – n (%) | 40 (98) | 34 (83) | 0.057 |  |
| Spironolactone – n (%) | 26 (63) | 16 (43) | 0.111 |  |
| LVEDV (ml) | 210±56 | 204±54 | 0.618 |  |
| LVESV (ml) | 153±52 | 151±50 | 0.848 |  |
| LVEF (%) | 28±7 | 28±7 | 0.982 |  |

*Legend: ACEi, angiotensin-converting-enzyme inhibitor; ARB, angiotensin-II receptor blocker; EDV, end-diastolic volume; EF, ejection fraction; ESV, end-systolic volume; ICM, ischemic cardiomyopathy; LBBB, left bundle branch block; LV, left ventricular; NYHA, New York Heart Association.*


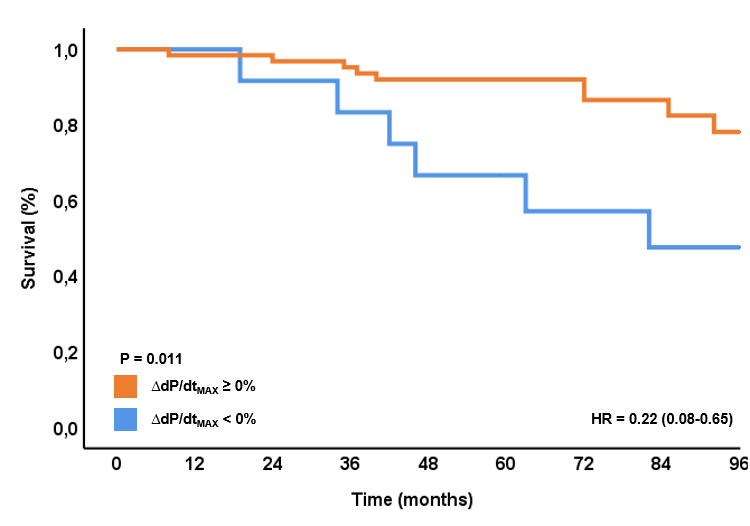


***Supplemental Figure 1.*** *Kaplan-Meijer curve for all-cause mortality, stratified according to acute change in LVdP/dt_MAX_.*
